# Two‐Dimensional Speckle Tracking Echocardiography and Real‐Time Three‐Dimensional Echocardiography in Marathon Runners: A Study of Left Atrium

**DOI:** 10.1002/clc.70230

**Published:** 2025-12-02

**Authors:** Pan Yang, Zhixiang Ge, Liping Wang, Haiyan Ke, Min Xu

**Affiliations:** ^1^ Department of Echocardiography of The Third Affiliated Hospital of Soochow University Changzhou First People's Hospital Changzhou City Jiangsu Province China; ^2^ Department of Cardiovascular Division of The Third Affiliated Hospital of Soochow University Changzhou First People's Hospital Changzhou City Jiangsu Province China

**Keywords:** left atrium, left ventricular hypertrophy, marathon, real‐time three‐dimensional echocardiography, two‐dimensional speckle tracking echocardiography

## Abstract

**Objective:**

To assess left atrial (LA) structure and function in marathon runners using two‐dimensional speckle tracking echocardiography (2D‐STE) and real‐time three‐dimensional echocardiography (RT‐3DE).

**Methods:**

This study enrolled 50 healthy volunteers (control group) and 132 marathon runners, and collected their general information. 2D‐STE and RT‐3DE were performed to obtain LA structural and functional parameters and left ventricular mass index (LVMI). According to the LVMI criteria for diagnosing left ventricular hypertrophy (LVH), all marathon runners were divided into an LVMI normal group and an LVH group. A comparative analysis was performed among the three groups. Multivariate logistic regression was used to analyze the association, and curve fitting was used to show the change trends.

**Results:**

Compared with the control group, LA total ejection fraction (LATEF) and LA passive ejection fraction (LAPEF) were higher in the LVMI normal group (*p* < 0.05). Compared with the control group and the LVMI normal group, LA maximal volume index (LAVImax), LA presystolic volume index (LAVIpre), and LA stiffness index (LASI) were higher in the LVH group, whereas LA reservoir strain (LASr), LA conduit strain (LAScd), and LA contraction strain (LASct) were lower (*p* < 0.05). Multivariate logistic regression analysis showed that LAVImax, LAScd, and LASct were significantly associated with LVH in marathon runners. Curve fitting showed that LAVImax increased with the increase of LVMI, whereas LAScd and LASct decreased.

**Conclusion:**

2D‐STE and RT‐3DE can effectively assess LA structure and function in marathon runners. Marathon runners with normal LVMI exhibit normal LA structure and function, and even some functional enhancement, while those with LVH exhibit increased LA volume and decreased LA strain function.

## Graphical abstract

Two‐dimensional speckle tracking echocardiography and real‐time three‐dimensional echocardiography can effectively assess left atrial structure and function in marathon runners.

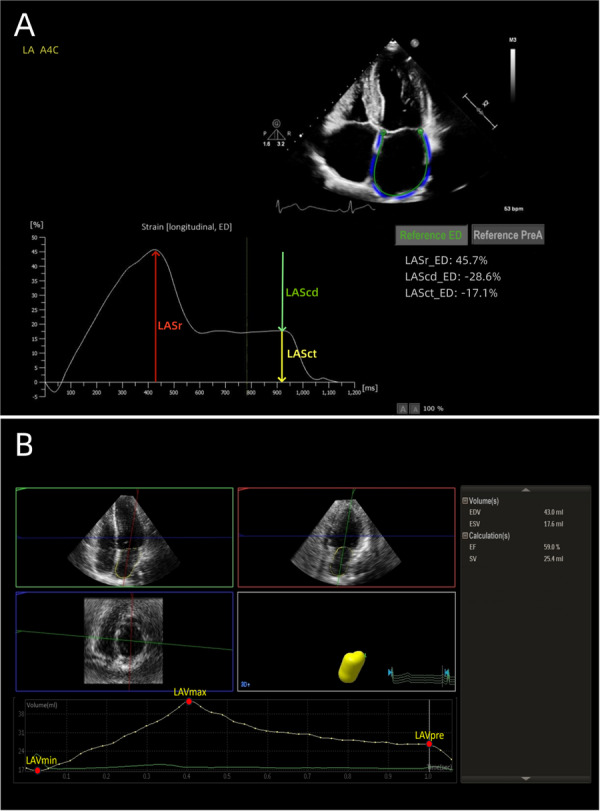

## 1 Introduction

In recent years, with the rising popularity of marathon running, there has been a growing number of marathon enthusiasts participating in the sport. During marathon running, due to the large amount of skeletal muscle movement, pulmonary ventilation and cardiac output increase dramatically, and the body's metabolic state changes significantly, which gradually leads to changes in cardiac hemodynamics and cardiac electrophysiology [1, 2]. The long‐term accumulation of these changes will cause physiological adaptation and remodeling of the heart, leading to the “athlete's heart,” including left ventricular hypertrophy (LVH), enlargement of the heart chambers, and a decrease in resting heart rate, and so forth [3, 4]. Left atrium (LA) is more susceptible to structural and functional changes than left ventricle (LV) due to its thinner wall and poorer ability to adapt to volume and pressure overload [5], which in turn causes various atrial arrhythmias, secondary LV changes, and even the occurrence of sudden cardiac death (SCD). Therefore, pre‐race and routine cardiac health assessment is particularly important for endurance athletes such as marathon runners. The development of ultrasound equipment and techniques has promoted the use of new ultrasound technologies, such as two‐dimensional speckle tracking echocardiography (2D‐STE) and real‐time three‐dimensional echocardiography (RT‐3DE), in sports medicine such as marathon.

Previous studies have focused more on LV structural and functional changes in marathon runners [6, 7], while there have been few studies on LA. And most of the studies on LA in patients with LVH have focused on diseases such as hypertension and hypertrophic cardiomyopathy [8, 9], while there have been few studies on endurance exercise‐related LVH. Therefore, this study intends to combine 2D‐STE and RT‐3DE to obtain LA parameters in marathon runners, use LVH as the grouping criterion, and contrast them with healthy individuals without a history of long‐term physical exercise. Our objective is to detect early LA structural and functional changes in marathon runners, which may be of significant importance for assessing the cardiac health of marathon runners and optimizing training programs.

## 2 Materials and Methods

### 2.1 Participants

This study was a prospective study that consecutively enrolled marathon runners in Changzhou City from September 2022 to December 2023. Inclusion criteria: age 18−70 years; marathon running age > 1 year, average monthly running distance > 60 km; having participated in “Class A1” marathon events certified by the Chinese Athletics Association [10] and met the standards. Exclusion criteria: cardiac insufficiency (LV ejection fraction [LVEF] < 50%) [11]; arrhythmia, coronary artery disease, congenital heart disease, valvular heart disease, cardiomyopathy, and history of cardiac surgery; metabolic diseases such as hypertension and diabetes mellitus; pulmonary, hepatic, renal, immunologic, hematologic, and psychiatric disorders; poor echocardiographic sound window transmission. A total of 150 runners were enrolled, and 132 were ultimately selected, including 85 males and 47 females, aged 28−65 years.

**Figure 1:**
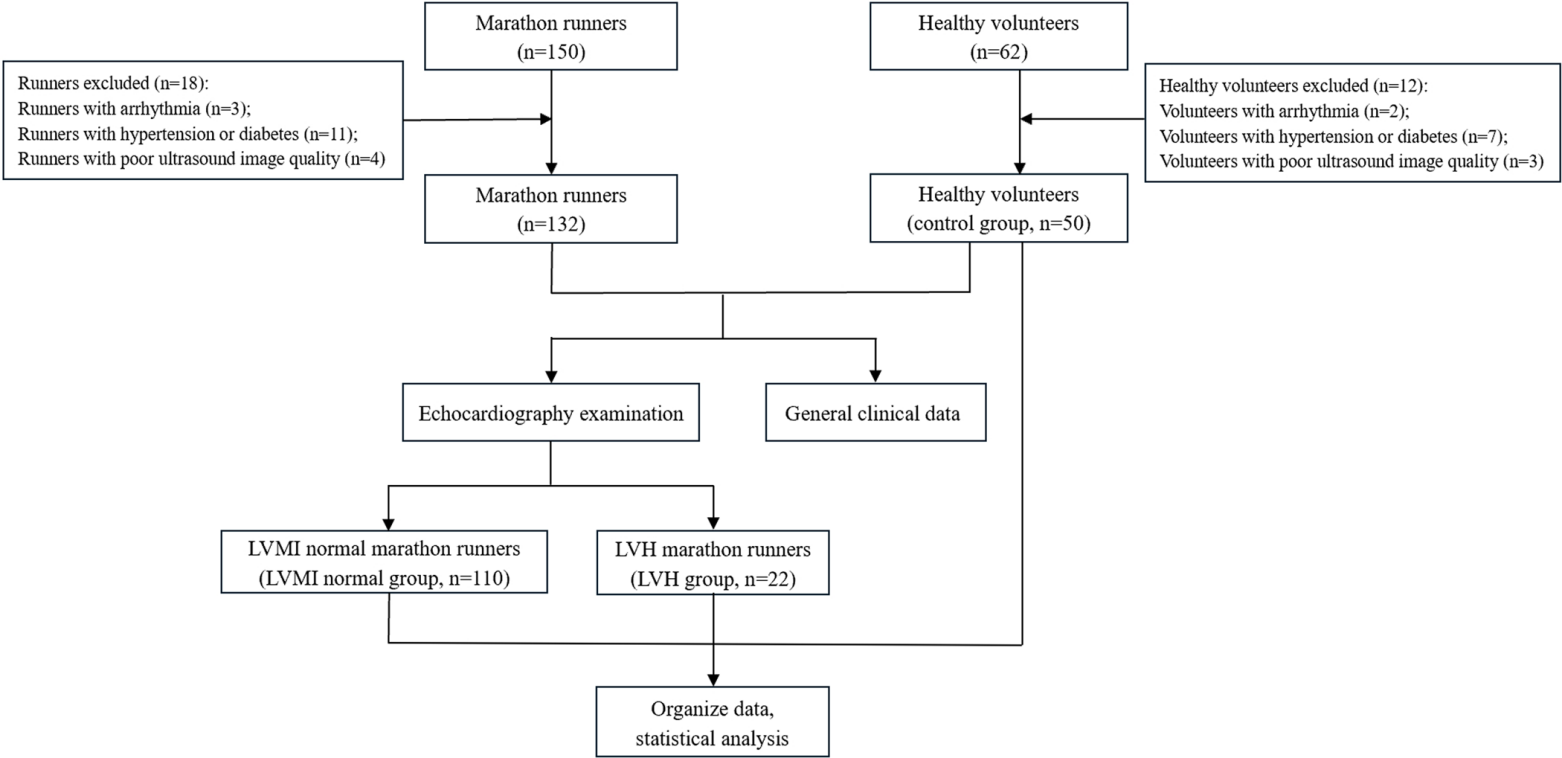
Flowchart of the study. LVH, left ventricular hypertrophy; LVMI, left ventricular mass index.

Concurrently, healthy volunteers who underwent echocardiography at Changzhou First People's Hospital were enrolled as the control group. Inclusion criteria: age 18−70 years; age, gender, and body mass index (BMI) distribution matching that of marathon runners; no history of long‐term physical exercise, with weekly physical activity duration < 2 h. Exclusion criteria: cardiac insufficiency, arrhythmia, coronary artery disease, congenital heart disease, valvular heart disease, cardiomyopathy, and history of cardiac surgery; metabolic diseases such as hypertension and diabetes mellitus; pulmonary, hepatic, renal, immunologic, hematologic, and psychiatric disorders; poor echocardiographic sound window transmission. A total of 62 volunteers were enrolled, and 50 were ultimately selected, including 31 males and 19 females, aged 28−64 years. This study was approved by the Medical Ethics Committee of our hospital (Ethics Approval No: 2022 Section No. 177), and all signed an informed consent form. The study flowchart is shown in Figure 1.

### 2.2 General Clinical Data

The data collected included age, height, weight, blood pressure, body surface area (BSA), BMI, smoking history, and alcohol consumption history of all participants. For marathon runners, additional data were collected, including family history of SCD, marathon running age, average monthly running distance, number of full marathons races, best time in full marathon races, number of half‐marathon races, and best time in half‐marathon races. Marathon running age was divided into three levels: < 3 years, 3−6 years and > 6 years, and average monthly running distance was divided into four levels: < 100 km, 100−200 km, 200−300 km and > 300 km.

### 2.3 Echocardiography Examination and Image Analysis

#### 2.3.1 Instrumentation

A Philips EPIQ 7C color Doppler echocardiographer equipped with S5‐1 and X5‐1 probes (Philips Healthcare Royal Philips Electronics, Amsterdam, Netherlands). The S5‐1 probe was used to obtain two‐dimensional transthoracic images at a frequency of 1.0−5.0 MHz, and the X5‐1 probe was used to obtain three‐dimensional transthoracic images at a frequency of 1.5−4.0 MHz. Image post‐processing was performed using QLab13.0 analysis software.

#### 2.3.2 Routine 2D Echocardiography and 2D‐STE Examination and Image Analysis

**Figure 2:**
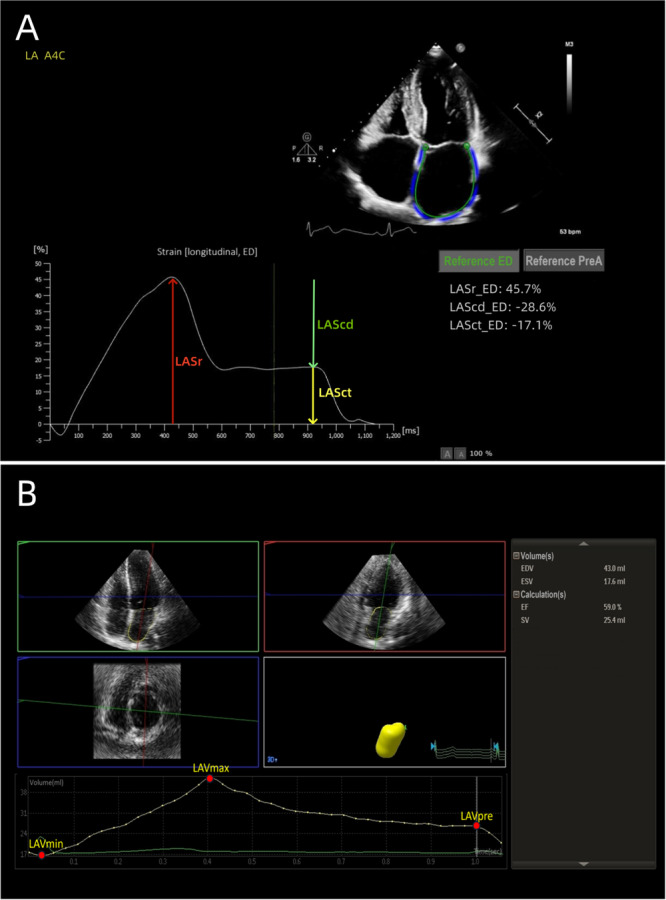
(A) LA 2D‐STE image. (B) LA RT‐3DE image. LAScd, left atrial conduit strain; LASct, left atrial contraction strain; LASr, left atrial reservoir strain; LAVmax, left atrial maximal volum; LAVmin, left atrial minimal volume; LAVpre, left atrial presystolic volume.

Participants should avoid strenuous exercise for 1 week before the examination. Participants were instructed to breathe calmly and lie in the left lateral position during the examination, and a limb‐lead ECG was connected simultaneously. 2D and color flow images of the standard parasternal long‐axis view, apical four‐chamber view, two‐chamber view, and three‐chamber view were obtained using the S5‐1 probe, and all images were obtained for at least three cardiac cycles. Routine measurements of early diastolic peak flow velocity (E) and late diastolic peak flow velocity (A) of the mitral orifice blood flow were obtained, as well as the mean of early diastolic tissue Doppler peak velocities of the lateral and septal sides of the mitral annulus (e'), and then calculated E/e'. Images were taken offline and imported into the analysis software, and LA reservoir strain (LASr), LA conduit strain (LAScd), and LA contraction strain (LASct) could be obtained automatically by using the automated measurement mode of LA strain (AutoStrain LA) and manually adjusting endocardial boundaries if necessary. LASr was positive, whereas LAScd and LASct were both negative [12] (Figure 2A), and data comparisons were based on their absolute values. LA stiffness index (LASI) is the ratio of LA pressure and reservoir strain, where LA pressure can be replaced by the parameter E/e’, that is, LASI = E/e’/LASr [13, 14].

#### 2.3.3 RT‐3DE Examination and Image Analysis

Switching to the X5‐1 probe, participants were instructed to hold their breath at the end of expiration, and real‐time three‐dimensional full‐volume mode was initiated in a standard apical four‐chamber view of the heart, and RT‐3DE images were obtained for at least three cardiac cycles. Images were taken offline and imported into the analysis software, and LVEF, LV mass (LVM), LA maximal volume (LAVmax), LA minimal volume (LAVmin), and LA presystolic volume (LAVpre, the corresponding time phase was the onset of the ECG P‐wave) were automatically obtained by using the automatic three‐dimensional quantization mode (3DQA) and manually adjusting endocardial and epicardial boundaries if necessary (Figure 2B). Then LVM index (LVMI), LAVmax index (LAVImax), LAVmin index (LAVImin) and LAVpre index (LAVIpre) were obtained by dividing by BSA. LA total ejection fraction (LATEF) was calculated as follow: LATEF = (LAVmax−LAVmin)/LAVmax, LA passive ejection fraction (LAPEF) was calculated as follow: LAPEF = (LAVmax−LAVpre)/LAVmax, and LA active ejection fraction (LAAEF) was calculated as follow: LAAEF = (LAVpre−LAVmin)/LAVmax.

### 2.4 Statistical Analysis

SPSS software (version 26.0) and R software (version 4.3.1, http://www.R-project.org/) were used for data analysis. Normally distributed measurement data were expressed as mean ± standard deviation (x ± s). A *t*‐test was used to compare two groups. A one‐way ANOVA was used to compare three groups, and a Bonferroni test was used for post hoc multiple comparisons; non‐normally distributed data were expressed as P50 (P25, P75). A Mann‐Whitney test was used to compare two groups, and a Kruskal−Wallis test was used to compare three groups. Count data were expressed as percentages (%), and Pearson's chi‐squared test or Fisher's exact test was used for categorical variables. Multivariate logistic regression analysis was used to identify 2D‐STE and RT‐3DE parameters that were significantly associated with LVH in marathon runners, and variance inflation factor (VIF) was used to assess multicollinearity among all the included parameters. Generalized additive model (GAM) was used to show the correlation and change trend between two variables. Statistical significance was determined using a two‐sided test, with a significance level set at *p* < 0.05.

## 3 Results

### 3.1 General Clinical Data

According to the standards of the American Society of Echocardiography (ASE), the diagnostic criteria for LVH are LVMI > 115 g/m^2^ for men and > 95 g/m^2^ for women [15]. All marathon runners were divided into an LVMI normal group (*n* = 110) and an LVH group (*n* = 22) based on the measured LVMI parameters. There were no statistically significant differences in age, sex, BSA, BMI, systolic blood pressure (SBP), diastolic blood pressure (DBP), smoking history, and alcohol consumption history among the LVMI normal group, the LVH group, and the healthy control group (all *p* > 0.05). Similarly, there were no statistically significant differences in family history of sudden death, running age of marathon, number of full marathons races, best time in full marathon races, number of half‐marathon races, and best time in half‐marathon races between the LVMI normal group and the LVH group (all *p* > 0.05). However, a statistically significant difference was observed between the two groups on average monthly running distance (*p* < 0.05). Compared with the LVMI normal group, the LVH group had a higher average monthly running distance (*p* < 0.05), as shown in Table 1.

**Table 1:** Comparison of general clinical data among the three groups.

Characteristics	control group (*n* = 50)	LVMI normal group (*n* = 110)	LVH group (*n* = 22)	*p* value
Age (year)	47.34 ± 7.88	45.58 ± 7.51	47.95 ± 7.11	0.228
Male (*n*, [%])	31 (62.0)	72 (65.5)	13 (59.1)	0.832
BSA (m^2^)	1.70 ± 0.17	1.71 ± 0.17	1.67 ± 0.14	0.528
BMI (kg/m^2^)	23.30 ± 2.55	22.50 ± 2.30	22.67 ± 1.81	0.131
SBP (mmHg)	125.20 ± 12.51	127.70 ± 14.21	128.77 ± 10.28	0.456
DBP (mmHg)	75.76 ± 7.59	76.95 ± 9.57	77.86 ± 11.24	0.627
Smoking history (*n*, [%])	7 (14.0)	16 (14.5)	2 (9.1)	0.862
Alcohol consumption history (*n*, [%])	11 (22.0)	30 (27.3)	4 (18.2)	0.574
Family history of sudden death (*n*, [%])	/	2 (1.8)	0 (0.0)	0.693
Running age of marathon (*n*, [%])	/			0.875
< 3 year		44 (40.0)	9 (40.9)	
3−6 year		41 (37.3)	7 (31.8)	
> 6 year		25 (22.7)	6 (27.3)	
Average monthly running distance (*n*, [%])	/			0.046
< 100 km		27 (24.5)	1 (4.5)	
100−200 km		41 (37.3)	11 (50.0)	
200−300 km		35 (31.8)	6 (27.3)	
> 300 km		7 (6.4)	4 (18.2)	
Number of half‐marathon races (time)	/	10 (3, 20)	9 (2, 15)	0.239
Best time in half‐marathon races (min)	/	102.77 ± 21.25	102.25 ± 10.92	0.916
Number of full marathon races (time)	/	2 (1, 7)	1 (1, 4.5)	0.368
Best time in full maratho races (min)	/	227.06 ± 44.64	242.77 ± 49.82	0.186

*Note:* Data were presented as mean ± SD or P50 (P25, P75) or *n* (%).

Abbreviations: BMI, body mass index; BSA, body surface area; DBP, diastolic blood pressure; LVMI, left ventricular mass index; LVH, left ventricular hypertrophy; SBP, systolic blood pressure.

### 3.2 Routine 2D Echocardiography, 2D‐STE, and RT‐3DE Parameters

There were no statistically significant differences in E, A, LVEF, and LAAEF among the three groups (all *p* > 0.05). However, statistically significant differences were observed among the three groups in e’, E/e’, LASr, LAScd, LASct, LASI, LVMI, LAVImax, LAVImin, LAVIpre, LATEF, and LAPEF (all *p* < 0.05). Compared with the control group, LATEF and LAPEF were higher in the LVMI normal group (all *p* < 0.05). Compared with the control group, E/e’, LVMI, LAVImax, LAVIpre, and LASI were higher in the LVH group, whereas e’, LASr, LAScd, and LASct were lower (all *p* < 0.05). Compared with the LVMI normal group, E/e’, LVMI, LAVImax, LAVImin, LAVIpre, and LASI were higher in the LVH group, whereas e’, LASr, LAScd, and LASct were lower (all *p* < 0.05), as shown in Table 2.

**Table 2:** Comparison of Routine 2D echocardiography, 2D‐STE, and RT‐3DE parameters among the three groups.

Parameters	control group (*n* = 50)	LVMI normal group (*n* = 110)	LVH group (*n* = 22)	*p* value
E (cm/s)	84.66 ± 15.95	87.22 ± 14.95	82.00 ± 14.87	0.274
A (cm/s)	70.36 ± 14.37	67.51 ± 15.51	73.36 ± 15.51	0.113
e’ (cm/s)	11.05 ± 2.07	10.76 ± 2.02	9.09 ± 2.06^a,b^	0.001
E/e'	7.77 ± 1.29	8.23 ± 1.23	9.26 ± 1.74^a,b^	< 0.001
LASr (%)	48.89 ± 8.33	45.92 ± 9.86	35.54 ± 7.64^a,b^	< 0.001
LAScd (%)	−31.58 ± 6.28	−30.11 ± 8.18	−22.99 ± 5.51^a,b^	< 0.001
LASct (%)	−17.33 ± 3.74	−15.82 ± 4.91	−12.55 ± 3.99^a,b^	< 0.001
LASI	0.17 ± 0.04	0.19 ± 0.06	0.28 ± 0.14^a,b^	< 0.001
LVEF (%)	66.72 ± 3.04	65.62 ± 2.52	65.23 ± 3.90	0.109
LVMI (g/m^2^)	82.87 ± 11.99	87.63 ± 12.18	116.77 ± 12.23^a,b^	< 0.001
LAVImax (mL/m^2^)	28.76 ± 7.16	30.27 ± 7.06	36.72 ± 10.32^a,b^	< 0.001
LAVImin (mL/m^2^)	12.24 ± 4.23	10.49 ± 3.71	13.94 ± 6.92^b^	0.001
LAVIpre (mL/m^2^)	19.02 ± 5.48	17.67 ± 5.37	22.72 ± 8.65^a,b^	0.001
LATEF (%)	57.84 ± 7.08	65.50 ± 8.95^a^	63.03 ± 10.27	< 0.001
LAPEF (%)	33.98 ± 8.07	41.58 ± 11.73^a^	38.65 ± 11.17	< 0.001
LAAEF (%)	23.86 ± 5.70	23.91 ± 9.86	24.38 ± 7.81	0.960

*Note:* Data were presented as mean ± SD. Compared with the control group, ^a^ *p* < 0.05; Compared with the LVMI normal group, ^b^ *p* < 0.05.

Abbreviations: 2D‐STE, two‐dimensional speckle tracking echocardiography; A, late diastolic peak flow velocity of the mitral orifice flow; E, early diastolic peak flow velocity of the mitral orifice flow; e’, the mean of early diastolic tissue Doppler peak velocities of the lateral and septal sides of the mitral annulus; LAAEF, left atrial active ejection fraction; LAPEF, left atrial passive ejection fraction; LAScd, left atrial conduit strain; LASct, left atrial contraction strain; LASI, left atrial stiffness index; LASr, left atrial reservoir strain; LATEF, left atrial total ejection fraction; LAVImax, left atrial maximal volume index; LAVImin, left atrial minimal volume index; LAVIpre, left atrial presystolic volume; LVEF, left ventricular ejection fraction; LVH, left ventricular hypertrophy; LVMI, left ventricular mass index; RT‐3DE, real‐time three‐dimensional echocardiography.

### 3.3 Multivariate Logistic Regression Analysis

**Figure 3:**
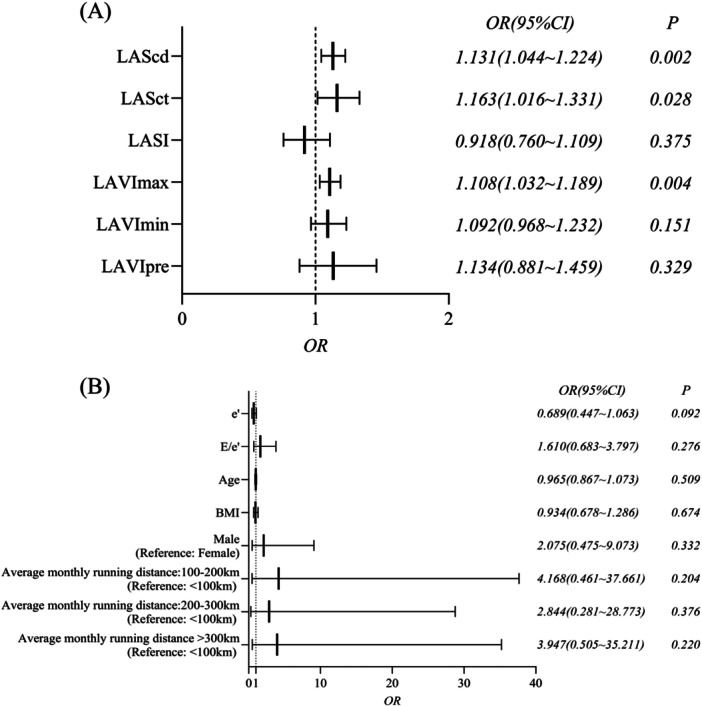
(A) Multivariate logistic regression analysis of 2D‐STE and RT‐3DE parameters. (B) Multivariate logistic regression analysis of potential confounders in the same model. 2D‐STE, two‐dimensional speckle tracking echocardiography; BMI, body mass index; E, early diastolic peak flow velocity of the mitral orifice flow; e’, the mean of early diastolic tissue Doppler peak velocities of the lateral and septal sides of the mitral annulus; LAScd, left atrial conduit strain; LASct, left atrial contraction strain; LASI, left atrial stiffness index; LAVImax, left atrial maximal volume index; LAVImin, left atrial minimal volume index; LAVIpre, left atrial presystolic volume; RT‐3DE, real‐time three‐dimensional echocardiography.

The occurrence of LVH was designated as the dependent variable, and all 2D‐STE and RT‐3DE parameters that were significantly different between the two groups were selected as the independent variables (LVMI was a subgrouping parameter, so it was excluded; LASr exhibited a high degree of collinearity with other independent variables, so it was also excluded). Multivariate logistic regression analysis showed that LAVImax (OR = 1.108, 95%CI = 1.032−1.189), LAScd (OR = 1.131, 95%CI = 1.044−1.224) and LASct (OR = 1.163, 95%CI = 1.016−1.331) were significantly associated with LVH in marathon runners after adjusting for potential confounders of sex, age, BMI, average monthly running distance, e’, and E/e’. The specific results of 2D‐STE and RT‐3DE parameters, along with potential confounding variables, were presented in Figures 3A,B, respectively.

### 3.4 GAM Test and Stratified Analysis

**Figure 4:**
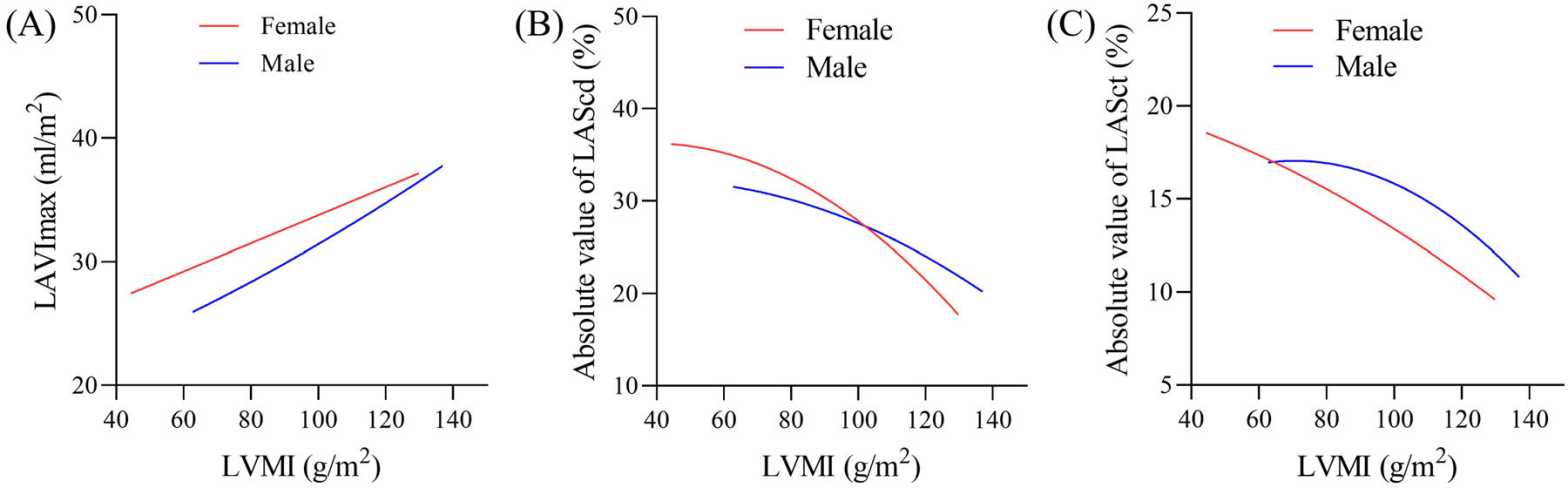
(A) Correlation and trend between LAVImax and LVMI in marathon runners. (B) Correlation and trend between LAScd and LVMI in marathon runners. (C) Correlation and trend between LASct and LVMI in marathon runners. LVMI, left ventricular mass index; LAVImax, left atrial maximal volume index; LAScd, left atrial conduit strain; LASct, left atrial contraction strain.

Curve fitting was used to test the correlations and trends between LAVImax, LAScd, LASct, and LVMI in marathon runners. Given that LVMI values used to diagnose LVH vary by gender, the analysis was stratified accordingly. The results showed that in marathon runners of different genders, LAVImax increased gradually with the increase of LVMI, and they were positively correlated (both degrees of freedom were close to 1, *p* < 0.05); LAScd and LASct all decreased gradually with the increase of LVMI, and they were negatively correlated (all degrees of freedom were close to 2, *p* < 0.05), as shown in Figure 4.

## 4 Discussion

Marathon is a high‐intensity endurance sport, which can result in LVH. Although this exercise‐induced LVH is different from the pathological LVH caused by diseases such as hypertension and aortic stenosis [16], this adaptive remodeling of the heart may also lead to adverse cardiac outcomes such as arrhythmias, cardiomyopathy, and even the development of SCD [17].

The interaction between LA and LV is central to maintaining normal left cardiac function and systemic circulation. Any functional abnormality in one directly impacts the other, forming an “interdependent and mutually influencing” relationship. This makes LA dimension and function closely associated with LV remodeling and function [18]. Furthermore, LA wall is much thinner than LV and is less adaptable to volume and pressure overload, and is more susceptible to enlargement and myocardial fibrosis during prolonged endurance exercise such as marathons [5], which affects cardiac hemodynamics and cardiac electrical conduction. Asif and coworkers [19] found that prolonged LA enlargement was associated with adverse cardiac events in athletes. LA enlargement and myocardial fibrosis also lead to the development of atrial arrhythmias, which are strongly associated with prognosis [20]. Flannery and coworkers [21] found that endurance athletes were more likely than the general population to develop atrial arrhythmias, particularly atrial fibrillation. Therefore, this study focuses on LA structural and functional changes in marathon runners.

During a complete cardiac cycle, LA undergoes three phases [22, 23]. The first phase is the reservoir phase, which occurs during LV systole and isovolumic diastole. During this phase, LA functions as a “reservoir” to receive blood from the pulmonary veins. This phase is mainly associated with LA compliance. The second phase is the conduit phase, which occurs in LV early diastole. This phase is LA passive emptying period, which is driven by a pressure gradient from LA to LV. The third phase is the contraction phase, which occurs in LV late diastole. This phase is LA active emptying period and associated with the intrinsic properties of LA myocardium. The degree of deformation of LA myocardium throughout the cardiac cycle can be expressed by the strains, that is, LASr, LAScd, and LASct, which are all negatively correlated with the degree of myocardial fibrosis [24]. LASr is associated with LA stiffness and compliance, which reflects LV diastolic function. LAScd is associated with LA and LV compliance. LASct is associated with LA active contractile ability and velocity, which is influenced by LA preload and afterload [25]. Furthermore, LA structural changes during the cardiac cycle can be quantified in terms of volumes, that is, LAVmax, LAVmin, and LAVpre.

With the development of ultrasound equipment and technology, the application of new technologies such as 2D‐STE and RT‐3DE in sports medicine has been promoted. Compared with traditional ultrasound techniques, 2D‐STE and RT‐3DE have no angle and volume dependence, can adapt to the anatomical positional differences when the heart chambers are enlarged, and can assess LA structure and function in a more detailed way [26, 27]. Therefore, in this study, we used 2D‐STE to obtain LA strain parameters, and RT‐3DE to obtain LA volume parameters and LVM.

In this study, we found that LATEF and LAPEF were higher in the LVMI normal group compared with the control group, and the remaining LA parameters did not show any obvious abnormality. This suggests that marathon runners with normal LVMI have normal LA structure and function, and even some functional enhancement, which may improve the function of the heart as a pump and the ability of the cardiovascular system to supply oxygen to the muscles during strenuous exercise. Furthermore, compared with the control group and the LVMI normal group, the LVH group had a higher average monthly running distance, and parameters E/e’, LAVImax, LAVIpre, and LASI were higher, whereas e’, LASr, LAScd, and LASct were lower. Among them, the decrease in e’ and the increase in E/e’ and LAVImax have been identified in guidelines as indicators of decreased LV diastolic function [28]. This suggests that marathon runners with LVH have increased LA volume and stiffness, decreased LA active contractility and compliance, and decreased LV diastolic function due to high‐intensity exercise loads. These LA changes may cause adverse cardiac events. Oxborough and coworkers [29] found that LA changes after prolonged, high‐intensity endurance exercise were correlated with decreased LV diastolic function, which is similar to the results of this study.

However, the parameters LVEF, LATEF, LAPEF, and LAAEF in the LVH group did not show significant differences compared with the other two groups, suggesting that marathon runners with LVH may have experienced early impairment of LA and LV function after prolonged marathon exercise, further validating the sensitivity of 2D‐STE and RT‐3DE. In this study, multivariate logistic regression analysis was further showed that LAVImax, LAScd, and LASct were significantly associated with LVH in marathon runners after adjusting for potential confounders, suggesting that LA volume and strain functional changes in marathon runners have a significant effect on LV. Curve fitting also showed that LAVImax gradually increased with the increase of LVMI, whereas LAScd and LASct all gradually decreased in marathon runners.

Therefore, marathon runners with LVH exhibit increased LA volume, decreased LA strain function, and LV diastolic function. This may be related to cardiac damage sustained from prolonged high‐intensity endurance exercise. During prolonged marathon training and LVH progression, LA compensates by enhancing myocardial contractility and enlarging its cavity to maintain necessary LV filling volume against increasing volume and pressure loads. However, sustained “overcompensation” eventually leads to LA decompensation, manifesting as irreversible LA enlargement and functional decline. Concurrently, this process activates inflammatory responses and neurohumoral systems (e.g., the renin‐angiotensin‐aldosterone system), increasing LA wall stiffness and myocardial fibrosis, and predisposing to various atrial arrhythmias. The altered LA, in turn, exacerbates LV pathology, creating a vicious cycle [5, 30]. Therefore, marathon runners with LVH may need to adjust their long‐distance training plans and marathon participation to reduce adverse cardiac events like arrhythmias or even SCD. For runners without LVH and the general population who enjoy long‐distance running, regular echocardiography is also important for detecting changes in LA and LV early, guiding appropriate exercise management.

There are some limitations of this study: firstly, the sample size of marathon runners participating in this study was small and a larger sample size is needed to validate the results; secondly, the influence of the right heart on LA function was neglected. In addition, cardiac magnetic resonance was not chosen to assess LVH because echocardiography is more applicable and common, given the outdoor complexity of the marathon, the high cost of magnetic resonance, and its prevalence in remote areas. Finally, the long‐term follow‐up of adverse cardiac events in marathon runners has not yet been completed.

In conclusion, 2D‐STE and RT‐3DE can effectively assess LA structure and function in marathon runners. Marathon runners with normal LVMI exhibit normal LA structure and function, and even some functional enhancement, while those with LVH exhibit increased LA volume and decreased LA strain function.

## Author Contributions

Pan Yang, Min Xu, participated in research design, data analysis, and interpretation, and writing of the manuscript; Zhixiang Ge, Liping Wang, participated in sample collection, and contributed to the acquisition and interpretation of data; Min Xu, Haiyan Ke, participated in research design and supervised the course of the project. All authors read and approved the final manuscript.

## Conflicts of Interest

The authors declare no conflicts of interest.

## Data Availability

The data underlying this article will be shared on reasonable request to the corresponding author.
